# Cytokine-Induced Loss of Glucocorticoid Function: Effect of Kinase Inhibitors, Long-Acting β2-Adrenoceptor Agonist and Glucocorticoid Receptor Ligands

**DOI:** 10.1371/journal.pone.0116773

**Published:** 2015-01-27

**Authors:** Christopher F. Rider, Suharsh Shah, Anna Miller-Larsson, Mark A. Giembycz, Robert Newton

**Affiliations:** 1 Airways Inflammation Research Group, Snyder Institute of Chronic Diseases, Faculty of Medicine, University of Calgary, Calgary, Alberta, Canada; 2 AstraZeneca R&D Mölndal, Mölndal, Sweden; Emory University, UNITED STATES

## Abstract

Acting on the glucocorticoid receptor (NR3C1), glucocorticoids are widely used to treat inflammatory diseases. However, glucocorticoid resistance often leads to suboptimal asthma control. Since glucocorticoid-induced gene expression contributes to glucocorticoid activity, the aim of this study was to use a 2×glucocorticoid response element (GRE) reporter and glucocorticoid-induced gene expression to investigate approaches to combat cytokine-induced glucocorticoid resistance. Pre-treatment with tumor necrosis factor-α (TNF) or interleukin-1β inhibited dexamethasone-induced mRNA expression of the putative anti-inflammatory genes RGS2 and TSC22D3, or just TSC22D3, in primary human airway epithelial and smooth muscle cells, respectively. Dexamethasone-induced DUSP1 mRNA was unaffected. In human bronchial epithelial BEAS-2B cells, dexamethasone-induced TSC22D3 and CDKN1C expression (at 6 h) was reduced by TNF pre-treatment, whereas DUSP1 and RGS2 mRNAs were unaffected. TNF pre-treatment also reduced dexamethasone-dependent 2×GRE reporter activation. This was partially reversed by PS-1145 and c-jun N-terminal kinase (JNK) inhibitor VIII, inhibitors of IKK2 and JNK, respectively. However, neither inhibitor affected TNF-dependent loss of dexamethasone-induced CDKN1C or TSC22D3 mRNA. Similarly, inhibitors of the extracellular signal-regulated kinase, p38, phosphoinositide 3-kinase or protein kinase C pathways failed to attenuate TNF-dependent repression of the 2×GRE reporter. Fluticasone furoate, fluticasone propionate and budesonide were full agonists relative to dexamethasone, while GSK9027, RU24858, des-ciclesonide and GW870086X were partial agonists on the 2×GRE reporter. TNF reduced reporter activity in proportion with agonist efficacy. Full and partial agonists showed various degrees of agonism on RGS2 and TSC22D3 expression, but were equally effective at inducing CDKN1C and DUSP1, and did not affect the repression of CDKN1C or TSC22D3 expression by TNF. Finally, formoterol-enhanced 2×GRE reporter activity was also proportional to agonist efficacy and functionally reversed repression by TNF. As similar effects were apparent on glucocorticoid-induced gene expression, the most effective strategy to overcome glucocorticoid resistance in this model was addition of formoterol to high efficacy NR3C1 agonists.

## Introduction

Acting via the glucocorticoid receptor (GR; NR3C1), glucocorticoids may reduce inflammatory gene expression by directly inhibiting the activity of inflammatory transcription factors (transrepression) and by increasing the transcription of genes (transactivation) with anti-inflammatory activity [1]. However, resistance to the anti-inflammatory effects of glucocorticoids can represent a major clinical challenge in many diseases. For example, while mild to moderate asthma is generally controlled by inhaled glucocorticoids (clinically known as inhaled corticosteroids (ICS)), glucocorticoid resistance is often present in more severe disease and during exacerbations [2,3]. In addition to substantial suffering and disability adjusted life years, individuals with severe, often poorly controlled, asthma command a disproportionately large share of health care expenditure [4,5]. Likewise, ICS are ineffective at reducing inflammation in the majority of individuals who smoke or have chronic obstructive pulmonary disease (COPD). Although not fully understood, mechanisms underlying glucocorticoid resistance may include increased P-glycoprotein-mediated efflux of glucocorticoid, increased expression of GRβ, an endogenous inhibitor of GR function, and reduced histone deacetylase-2 expression leading to decreased repression of inflammatory genes [2,3]. However, glucocorticoid activity is also reduced by pro-inflammatory cytokines, such as tumor necrosis factor α (TNF) and interleukin-1β (IL1B) [3,6,7].

There are a number of potential approaches to overcoming glucocorticoid resistance: 1) increase the glucocorticoid dose; 2) reverse the resistance by inhibiting inflammatory signaling pathways; 3) identify glucocorticoids, or other NR3C1 ligands, that are not subject to resistance; and, 4) potentiate glucocorticoid activity using long-acting β_2_-adrenoceptor agonists (LABAs) [2,3,8]. Alternatively, while other broad spectrum anti-inflammatory agents may theoretically be useful, those developed to date, including calcineurin inhibitors, methotrexate and phospodiesterase-4 inhibitors, have proved ineffective in the treatment of glucocorticoid-refractory asthma due to poor efficacy or undesirable side-effect profiles [2,9]. Likewise, although increasing dose or using oral corticosteroids is somewhat effective in asthma, this increases the risk of side effects, including diabetes, cataracts and osteoporosis [10]. Furthermore, higher glucocorticoid concentrations have little effect in an *in vitro* model of glucocorticoid resistance [6]. A better approach to overcoming glucocorticoid resistance may therefore be the inhibition of inflammatory pathways. For example, mitogen-activated protein kinase (MAPK), protein kinase C (PKC) and phosphoinositide 3-kinase (PI3K) pathways are all activated by TNF and have been implicated in the induction of glucocorticoid resistance [2,11–13]. Targeted inhibition of such pathways may therefore reverse the glucocorticoid hyporesponsiveness induced by TNF.

Ideally, glucocorticoid resistance could be overcome by identifying, if possible, novel NR3C1 ligands that are not susceptible to the mechanisms of resistance. However, in generating new glucocorticoids, pharmaceutical companies have focused on reducing side effects, producing compounds with differing potency, efficacy and metabolism [14,15]. Many ICS, including budesonide, fluticasone propionate and fluticasone furoate, undergo rapid hepatic deactivation to reduce systemic exposure [16]. Other ICSs, including ciclesonide, beclomethasone dipropionate and butixocort 21-propionate, are pro-drugs that are predominantly activated in the lung, thereby reducing local and systemic side effects resulting from the large fraction that is swallowed [14,15]. Non-steroidal NR3C1 ligands, including GSK9027, have also been developed [17]. Because of prevailing dogma that transrepression accounts for the benefits and transactivation for the side effects of glucocorticoids, pharmaceutical companies have endeavored to develop ‘dissociated’ NR3C1 ligands, which induce transrepression, but not transactivation [14,18]. These compounds, known as selective glucocorticoid receptor agonists (SEGRAs), include RU24858 and GW870086X [19,20]. However, many SEGRAs can induce transactivation and the enhancement of gene expression also mediates key anti-inflammatory actions of glucocorticoids [10,18,21,22]. Nevertheless, despite the many novel NR3C1 ligands available, it is currently unclear whether any can escape the effects of resistance.

A further approach to combating glucocorticoid resistance could be to enhance glucocorticoid activity. Thus, the addition of a LABA potentiates the effects of ICS therapy in asthma, providing greater symptom control than doubling, or even quadrupling, the glucocorticoid dose [8]. Furthermore, LABAs enhance NR3C1-dependent transcription and, by increasing the expression of glucocorticoid-inducible effector genes, counteract the loss of glucocorticoid function mediated by TNF or the synthetic double stranded RNA virus mimetic, polyinosinic-polycytidylic acid [6,23,24].

Given that glucocorticoid-inducible gene expression is up-regulated in asthmatics taking ICS [25], we have investigated, in primary human bronchial epithelial (HBE) and airways smooth muscle (ASM) cells, whether inflammatory cytokines reduce expression of genes with anti-inflammatory or anti-asthma properties. Our hypothesis was that inflammatory stimuli, such as TNF, reduce the expression of genes with putative anti-inflammatory or anti-asthma properties and that such effects may be reversed. For example, regulator of G-protein signaling 2 (RGS2) reduces inflammatory Gα_q_-linked signaling and is bronchoprotective *in vivo* [26–29]. Likewise, dual-specificity phosphatase 1 (DUSP1; MKP1) and cyclin-dependent kinase inhibitor 1c (CDKN1C; p57KIP2) inhibit either all mitogen-activated protein kinases (MAPKs) or the JNK MAPK pathway respectively [22,30,31], while TSC22 domain family protein 3 (TSC22D3; GILZ) decreases the activity of activator protein 1 and nuclear factor kappa-light chain-enhancer of activated B cells (NF-κB) [32]. We evaluated; i) the ability of NF-κB, MAPK, PI3K or PKC inhibitors to reverse TNF-induced glucocorticoid hyporesponsiveness, and ii) whether various steroidal and non-steroidal NR3C1 ligands were also affected by TNF-induced repression of dexamethasone-inducible gene expression, in the absence and presence of the LABA formoterol.

## Materials and Methods

### Cell Culture and Drugs

Human bronchial epithelial (BEAS-2B) cells (ATCC, Manassas, VA) [33] were cultured in Dulbecco’s modified Eagle’s/Ham’s F12 medium supplemented with 14 mM NaHCO_3_, 2 mM L-glutamine and 10% fetal calf serum (all from Life Technologies; Burlington, ON). Primary human ASM and HBE cells were isolated from non-transplanted normal human lungs obtained using the tissue retrieval service at the International Institute for the Advancement of Medicine, Edison, NJ, USA [23,34]. Individual hospital sites obtained consent from next of kin for the tissues to be used for either transplant or for research and the lungs were received without any unique patient identifiers. Ethics approval for this was obtained from both the Conjoint Health Research Ethics Board at the University of Calgary and the Internal Ethics Board of the International Institute for the Advancement of Medicine. ASM cells were cultured in Dulbecco’s modified Eagle’s medium supplemented with 10% fetal calf serum, 2 mM L-glutamine, 20 μg/ml penicillin/streptomycin and 2.5 μg/ml amphotericin B (Life Technologies) and used for experiments at passages 3 to 8. HBE cells were cultured in bronchial epithelial cell growth medium (BEGM; Lonza, Allendale, NJ) as previously described [34]. All cells were cultured at 37°C in 5% CO_2_/95% air and were incubated in serum- or additive-free medium overnight prior to experiments. TNF and IL1B (R&D systems; Minneapolis, MN) were dissolved in phosphate-buffered saline containing 0.1% bovine serum albumin (Sigma-Aldrich; St. Louis, MO). Dexamethasone (Sigma-Aldrich) was dissolved in Hank’s balanced salt solution (Life Technologies). Budesonide and formoterol (AstraZeneca, Sweden), des-ciclesonide (Takeda Pharmaceuticals International; Deerfield, IL), PS-1145 (Millennium Pharmaceuticals, Cambridge, USA), fluticasone furoate, fluticasone propionate, GW870086X (GlaxoSmithKline; Middlesex, UK), GF109203X, Gö6976, JNK inhibitor VIII, PD098059, SB203580 (Calbiochem/EMD Chemicals/Merck KGaA; Darmstadt, Germany), GSK9027, PI103 (Tocris Bioscience; Bristol, UK), LY294002, wortmannin (Sigma-Aldrich), Ro 31–8220 (Cayman Chemical Company; Ann Arbor, MI) and RU24858 (Sanofi-Aventis Pharmaceuticals; Dagenham, UK) were dissolved in dimethyl sulphoxide (Sigma-Aldrich). Final dimethyl sulphoxide concentrations on cells were ≤ 0.1%.

### Luciferase Reporter Assays

BEAS-2B cells stably transfected with a glucocorticoid response element (2×GRE) driven luciferase reporter plasmid, pGL3.neo.TATA.neo [23], were cultured in medium containing 0.25 mg/ml G-418 (Promega, Madison, WI). Cells were grown to confluence in 24 or 48 well plates and incubated overnight in serum-free media prior to experiments. Cells were harvested in reporter lysis buffer and luciferase assays performed according to the manufacturer’s instructions (Firefly Luciferase Assay Kit; Biotium; Hayward, CA) using a 20/20n luminometer (Promega).

### Real-Time PCR analysis

Total RNA was extracted using RNeasy Mini Kits (Qiagen; Valencia, CA) and 0.5 μg used for cDNA synthesis using the qScript cDNA synthesis kit (Quanta; Gaithersburg, MD). Following a 1:4 dilution, real-time PCR was performed on 2.5 μl of cDNA in 10 μl reactions using a 7900HT instrument (Applied Biosystems; Foster City, CA) with SYBR GreenER chemistry (Life Technologies). Amplification conditions of 50°C, 2 min; 95°C, 10 min; then 40 cycles of 95°C, 15 s; 60°C, 1 min were used, before melt curve analysis to confirm primer specificity. Primer sequences were: TSC22D3, forward 5′-GGC CAT AGA CAA CAA GAT CG-3′, reverse 5′-ACT TAC ACC GCA GAA CCA CCA-3′; CDKN1C, forward 5′-CGG CGA TCA AGA AGC TGT C-3′, reverse 5′-GGC TCT AAA TTG GCT CAC CG-3′; DUSP1, forward 5′-GCT CAG CCT TCC CCT GAG TA-3′, reverse 5′-GAT ACG CAC TGC CCA GGT ACA-3′; RGS2, forward 5′-CCT CAA AAG CAA GGA AAA TAT ATA CTG A-3′, reverse 5′-AGT TGT AAA GCA GCC ACT TGT AGC T-3′; and glyceraldehyde 3-phosphate dehydrogenase (GAPDH), forward 5′-TTC ACC ACC ATG GAG AAG GC-3′, reverse 5′-AGG AGG CAT TGC TGA TGA TCT-3′.

### Western Blotting

Cells were lysed in 1× Laemmli buffer supplemented with phosphatase inhibitors (Sigma-Aldrich) and 1× complete protease inhibitor cocktail (Roche; Indianapolis, IN), size fractionated on 12% acrylamide gels and subsequently electro-transferred onto Hybond enhanced chemiluminescent membranes (GE Healthcare; Waukesha, WI). Membranes were blocked in 5% milk in tris-buffered saline containing 1% tween 20 and probed with antibodies against phospho-jun proto-oncogene (pJUN; pc-Jun) (9164; Cell Signaling Technology; Danvers, MA), nuclear factor of kappa light polypeptide gene enhancer in B-cells inhibitor, alpha (NFKBIA; IκBα) (sc-371; Santa Cruz Biotechnology; Dallas, TX), phospho-NFKBIA (9246; Cell Signaling Technology), phospho-AKT1 (9271S; Cell Signaling Technology), AKT (9272S; Cell Signaling Technology) and GAPDH (4699–9555(ST); AbD Serotec; Raleigh, NC). After washing, membranes were incubated with horseradish peroxidase-conjugated anti-rabbit (111–035–003; Jackson ImmunoResearch Laboratories Inc; West Grove, PA) or anti-mouse immunoglobulin (115–035–003; Jackson ImmunoResearch Laboratories Inc). Detection was by enhanced chemiluminescence (Pierce ECL Western Blotting Substrate; Thermo Fisher Scientific Inc.; Rockford, IL) and visualization by autoradiography.

### Statistical Analysis

All data are plotted as means ± SE. Statistical analyses were performed using Prism version 6.01 (GraphPad Software, San Diego, CA). Student’s t test or analysis of variance (ANOVA) with Bonferroni’s correction for multiple comparisons or a Dunnett’s post test were used as indicated.

## Results

### Effects of TNF on Dexamethasone-Induced Gene Expression in Primary Human Bronchial Epithelial, Airway Smooth Muscle and BEAS-2B Cells

**Fig 1:**
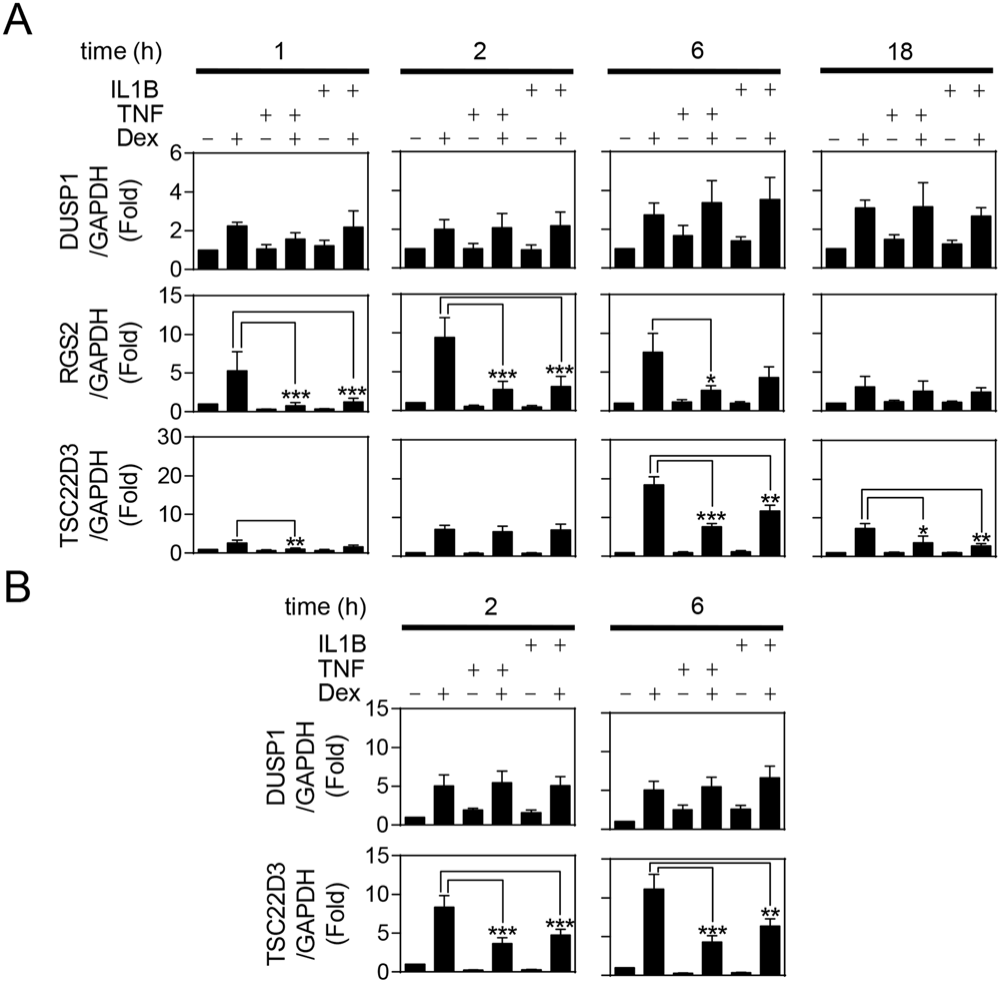
Effects of TNF and IL1B on dexamethasone-inducible gene expression in primary human structural lung cells. A. Primary human bronchial epithelial (HBE) cells were pre-treated for 1 h with 10 ng/ml of tumor necrosis factor-α (TNF) or 1 ng/ml of interleukin 1β (IL1B), before addition of 1 μM dexamethasone (Dex). Cells were harvested at 1, 2, 6 and 18 h after Dex addition. B. Airway smooth muscle (ASM) cells were pre-treated for 1 h with 10 ng/ml of TNF or 1 ng/ml of IL1B, before addition of 1 μM Dex. Cells were harvested at 2 and 6 h after Dex addition. Total RNA was extracted, reverse transcribed to cDNA and RT-PCR performed for: regulator of G-protein signaling 2 (RGS2), TSC22 domain family member 3 (TSC22D3; GILZ), dual specificity phosphatase 1 (DUSP1; MKP1) and glyceraldehyde-3-phosphate dehydrogenase (GAPDH). Data (n = 4–5), normalized to GAPDH, are expressed as fold and plotted as means ± S.E. Significance was tested using repeated measures, one-way analysis of variance (ANOVA) with Bonferroni’s correction for multiple comparisons. *, *P*<0.05; **, *P*<0.01; ***, *P*<0.001.

Primary HBE cells were pre-treated for 1 h with maximally effective concentrations of TNF or IL1B [6], before the addition of 1 μM dexamethasone (Fig. 1A). Dexamethasone enhanced DUSP1 mRNA expression at all time points tested (1, 2, 6 and 18 h) and this was not significantly affected by cytokine pre-treatment (Fig. 1A). RGS2 mRNA induction was also increased by dexamethasone, with expression peaking at 2 h and remaining high at 6 h, before decreasing at 18 h. However, pre-treatment with TNF or IL1B almost completely attenuated RGS2 expression at 1 h, reducing its level to that of unstimulated cells. This level of cytokine-induced repression was attenuated at 2 and 6 h, and by 18 h neither cytokine had any obvious effect on inducibility by dexamethasone. While TNF and IL1B suppressed dexamethasone-induced TSC22D3 expression at 1, 6 and 18 h, with maximal repression of 60 and 62% occurring at 1 h and 18 h for TNF and IL1B respectively, neither cytokine repressed TSC22D3 expression at 2 h. CDKN1C was not glucocorticoid inducible in HBE cells (data not shown).

In primary human ASM cells, pre-treatment with either TNF or IL1B had no effect on dexamethasone-induced DUSP1 mRNA expression (Fig. 1B). However, cytokine pre-treatment significantly reduced TSC22D3 expression at 2 and 6 h, with repressions of 61 and 43% produced by TNF and IL1B respectively at 6 h. Expression of CDKN1C and RGS2 was not materially increased by dexamethasone in ASM cells (data not shown).

**Fig 2:**
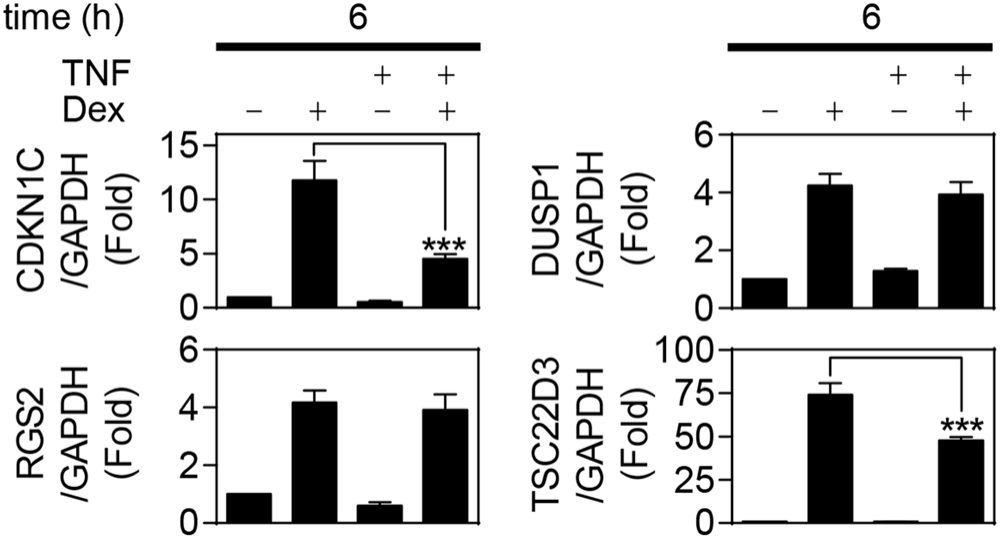
Effect of TNF on dexamethasone-induced gene expression in BEAS-2B cells. Human bronchial epithelial, BEAS-2B, cells were pre-treated for 1 h with 10 ng/ml of tumor necrosis factor-α (TNF), before addition of 1 μM dexamethasone (Dex). Cells were harvested 6 h after Dex addition. Total RNA was extracted, reverse transcribed to cDNA and RT-PCR performed for: cyclin-dependent kinase inhibitor 1C (CDKN1C; p57KIP2), dual specificity phosphatase 1 (DUSP1; MKP1), regulator of G-protein signaling 2 (RGS2), TSC22 domain family member 3 (TSC22D3; GILZ) and glyceraldehyde-3-phosphate dehydrogenase (GAPDH). Data (n = 7), normalized to GAPDH, are expressed as fold and plotted as means ± S.E. Significance was tested using repeated measures, one-way analysis of variance (ANOVA) with Bonferroni’s correction for multiple comparisons. ***, *P*<0.001.

To evaluate the BEAS-2B human bronchial epithelial cell line as a model for cytokine-induced glucocorticoid resistance, the effect of TNF was tested on the dexamethasone-induced expression of DUSP1, CDKN1C, RGS2 and TSC22D3 at 6 h. As described for both the HBE and ASM cells, TNF pre-treatment did not affect dexamethasone-induced DUSP1 expression (Fig. 2). Dexamethasone substantially induced CDKN1C expression in BEAS-2B cells, but this was attenuated by 62% following TNF treatment. RGS2 was also induced by dexamethasone treatment and, in contrast to the HBE cells, TNF pre-treatment had no effect. Conversely, dexamethasone induced TSC22D3 expression by 74 fold and, as described in the HBE and ASM cells, this was significantly repressed following TNF pre-treatment (Fig. 2). Collectively, these data indicate that cytokines induce glucocorticoid hyporesponsiveness in a cell-, gene- and time-dependent manner and that the BEAS-2B cell line may be used as a relevant model of this cytokine-mediated repression.

### Effect Of MAPK and NF-Κb Inhibitors on TNF-Induced Glucocorticoid Resistance

**Fig 3:**
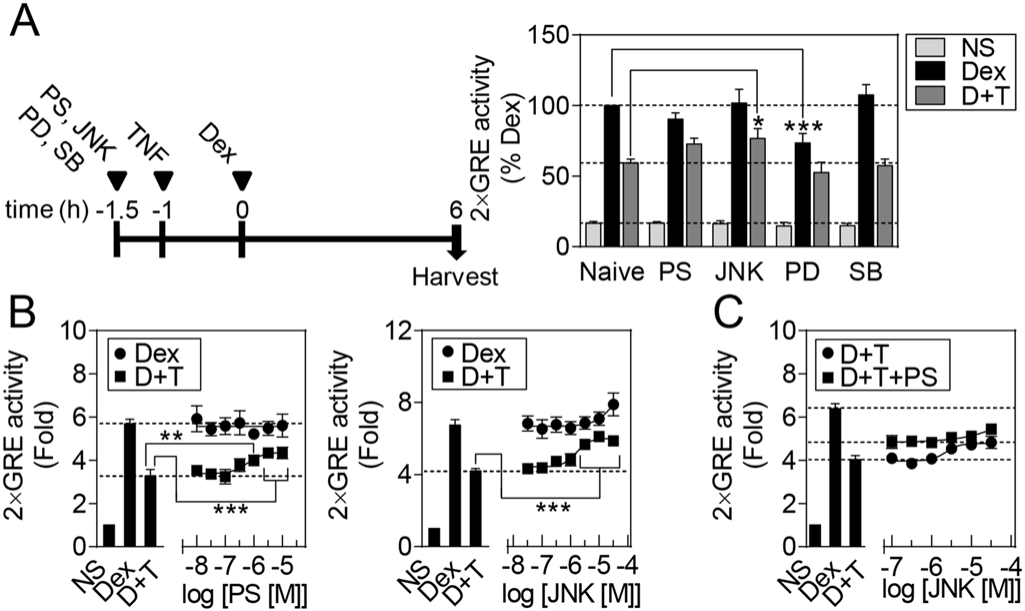
Effects of IKK2 and MAPK inhibitors on repression of dexamethasone-induced 2×GRE reporter activation by TNF. A. BEAS-2B cells stably transfected with a 2×glucocorticoid response element (GRE) luciferase reporter were pre-treated for 30 min with 10 μM of the NF-κB or MAPK pathway inhibitors: PS-1145 (PS; IKBKB), PD098059 (PD; MAP2K1/2), SB203580 (SB; p38 MAPKs) or JNK inhibitor VIII (JNK; JNK MAPKs), before addition of 10 ng/ml of tumor necrosis factor-α (TNF). After 1 h, 10 μM dexamethasone (Dex) was added and cells harvested 6 h later for luciferase assay. Data (n = 7), expressed as a percentage of Dex activation, are plotted as means ± S.E. BEAS-2B 2×GRE cells were pre-treated for 30 min with the indicated concentrations of B. PS or JNK or C. JNK in the presence or absence of 10 μM PS, before addition of 10 ng/ml TNF. After 1 h, 10 μM Dex was added and cells harvested 6 h later for luciferase assay. Data (n = 4–8), expressed as fold activation, are plotted as means ± S.E. Significance was tested using repeated measures, one-way analysis of variance (ANOVA) with Bonferroni’s correction for multiple comparisons. *, *P*<0.05; **, *P*<0.01; ***, *P*<0.001. D+T indicates Dex plus TNF treatment.

BEAS-2B cells harboring a 2×GRE luciferase reporter were pre-treated for 30 min with maximally effective concentrations of the inhibitors PS-1145, JNK inhibitor VIII, PD098059 or SB203580, prior to TNF treatment [22,28,35,36]. Dexamethasone was added 1 h after TNF treatment and luciferase activity determined 6 h later (Fig. 3A). Dexamethasone induced reporter activity and this was reduced by 41% in the presence of TNF (Fig. 3A). Pre-treatment with the IκB kinase 2 (IKK2) inhibitor, PS-1145 (10 μM), restored reporter induction to 73% of that induced by dexamethasone. Similarly, JNK inhibitor VIII [37], also partially reversed the TNF-induced repression and restored reporter activity to 77% of the level induced by dexamethasone alone. Conversely, the MAPK kinase 1/2 (MAP2K1/MAP2K2) inhibitor, PD098059 [38], significantly reduced dexamethasone-induced 2×GRE reporter activation, while the p38 MAPK inhibitor, SB203580 [39], had no effect on dexamethasone-induced reporter activation, either in the absence, or presence, of TNF.

To evaluate the effect of PS-1145 and JNK inhibitor VIII, on their respective targets, western blotting was performed for the phosphorylated forms of NFKBIA (IκBα) and JUN (c-Jun) (S1 Fig.). PS-1145 concentration-dependently reduced NFKBIA phosphorylation induced by TNF with an EC_50_ of ~1 μM. Likewise, JNK inhibitor VIII decreased TNF-induced JUN phosphorylation in a concentration-dependent manner (EC_50_ ~0.3 μM) (S1 Fig.). The effect of various concentrations of PS-1145 or JNK inhibitor VIII were evaluated on the 2×GRE reporter (Fig. 3B). PS-1145 had no effect on dexamethasone-induced reporter activation, but reversed (EC_50_ 0.6 μM) the TNF-induced repression from ~60% to 76% of the maximum produced by dexamethasone. Likewise, JNK inhibitor VIII reversed (EC_50_ 1.6 μM) the TNF-induced repression to 87% of the response to dexamethasone (Fig. 3B). Finally, while JNK inhibitor VIII modestly enhanced dexamethasone-induced reporter activation, this was only apparent at 30 μM of JNK and may therefore represent an off-target effect.

To examine whether simultaneous inhibition of IKK2 and JNK may combine to fully reverse the repression of glucocorticoid activity by TNF, various concentrations of JNK inhibitor VIII were tested in the absence and presence of a maximally effective concentration (10 μM) of PS-1145 (Fig. 3C). However, at up to 10 μM of JNK inhibitor VIII, there was no obvious additional effect of PS-1145 on 2×GRE reporter activity.

### Effect of Inhibitors of Phosphoinositide 3-Kinase and Protein Kinase C on TNF-Induced Glucocorticoid Hyporesponsiveness

**Fig 4:**
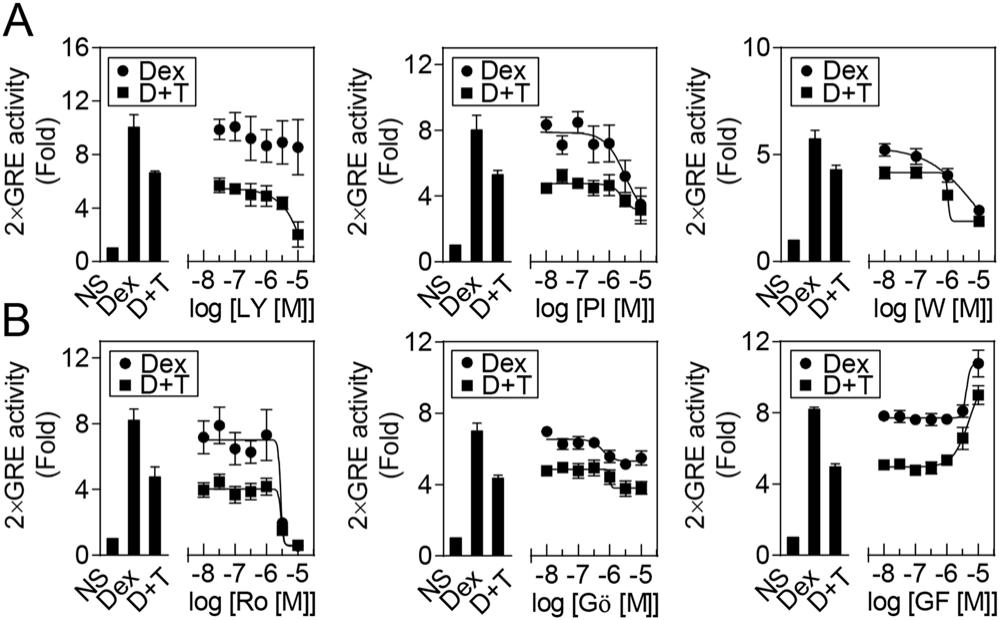
Effects of PKC or PI3K inhibitors on TNF-mediated repression of dexamethasone induced 2×GRE reporter activation. BEAS-2B 2×GRE cells were pre-treated for 30 min with the indicated concentrations of A. the phosphoinositide 3-kinase (PI3K) inhibitors: LY294002 (LY), PI103 (PI) and Wortmannin (W); or B. the protein kinase C (PKC) inhibitors: Ro 31–8220 (Ro), Gö6976 (Gö) and GF109203X (GF); before addition of 10 ng/ml of tumor necrosis factor-α (TNF). After 1 h, 10 μM dexamethasone (Dex) was added and cells harvested 6 h later for luciferase assay. Data (n = 3–4), expressed as fold activation, are plotted as means ± S.E. D+T indicates Dex plus TNF treatment.

As inhibition of PI3K can reduce glucocorticoid resistance [40,41], we investigated the effects of the PI3K inhibitors LY294002, PI103 and wortmannin on TNF-induced repression of glucocorticoid activity [42–44]. All three inhibitors blocked AKT1 phosphorylation, a downstream target of PI3K, at concentrations lower than 10 μM, with EC_50_ values of ~900, 70 and 20 nM for LY294002, PI103 and wortmannin respectively (S2 Fig.). Both PI103 and wortmannin concentration-dependently reduced the 2×GRE reporter activation induced by dexamethasone, while LY294002 had less effect (Fig. 4A). In the presence of TNF, all three compounds further reduced reporter activation induced by dexamethasone. These data do not support a role for PI3K in the reduction of dexamethasone-induced reporter activity by TNF.

To investigate the effect of PKC inhibition, BEAS-2B cells were pre-treated for 30 min with various concentrations of Ro31–8220, Gö6976 or GF109203X, before the addition of TNF and 1 h later dexamethasone (Fig. 4B) [45–47]. Below concentrations of 1 μM, there was no effect of these inhibitors on 2GRE reporter activation induced by dexamethasone or the repression produced by TNF. However, above 1 μM, both Ro 31–8220 and Gö6976 reduced dexamethasone-induced reporter activity. Conversely, at concentrations of over 1 μM, GF109203X enhanced dexamethasone-induced 2×GRE reporter activation both in the absence and presence of TNF. These data suggest that there is no clear role for PKC in the repression of dexamethasone-induced reporter activation by TNF.

### Effect of Different NR3C1 Ligands and Formoterol on TNF-Induced Glucocorticoid Resistance

**Fig 5:**
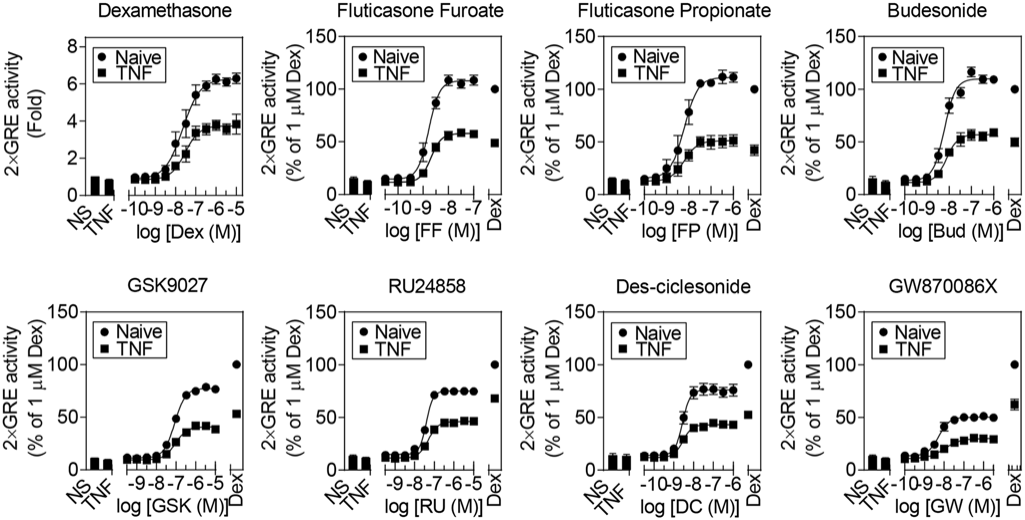
Effects of TNF on 2×GRE reporter activation induced by NR3C1 ligands. BEAS-2B 2×GRE cells were pre-treated with 10 ng/ml of TNF for 1 h, before addition of NR3C1 ligands: the glucocorticoids dexamethasone (Dex), fluticasone furoate (FF), fluticasone propionate (FP), budesonide (Bud) and des-ciclesonide (DC); the non-steroidal NR3C1 agonist GSK9027 (GSK) or the selective glucocorticoid receptor agonists (SEGRAs): RU24858 (RU) and GW870086X (GW), at the indicated concentrations. Cells were harvested 6 h after NR3C1 ligand addition for luciferase assay. Data (n = 4–6), expressed as fold activation (Dex) or as a percentage of Dex activation, are plotted as means ± S.E. Statistical significance was tested using repeated measures, one-way analysis of variance (ANOVA) with Bonferroni’s correction for multiple comparisons. At least the top four concentrations of each NR3C1 ligand had statistical significance of *P*<0.001 (***) relative to the NR3C1 ligand in the presence of TNF.

To examine the susceptibility of structurally dissimilar NR3C1 ligands to repression by TNF, the concentration-response relationships of various steroidal and non-steroidal NR3C1 agonists were examined in the absence and presence of TNF (Fig. 5). Relative to a maximally effective concentration (1 μM) of dexamethasone, these ligands produced a range of maximal responses (*E*_Max_). Fluticasone furoate, fluticasone propionate and budesonide all produced similar *E*_Max_ values to dexamethasone (Table 1). By comparison, RU24858, des-ciclesonide, GSK9027 and, in particular, GW870086X revealed reduced *E*_Max_ values relative to dexamethasone. TNF pre-treatment reduced the maximal response produced by each ligand and this effect was not surmountable by high ligand concentrations (Fig. 5). In addition to reducing the *E*_Max_ following TNF treatment, there was also a trend, significant for dexamethasone, RU24858 and GW870086X, towards modestly reduced agonist potency (Table 1). However, this difference in agonist potency is of questionable biological significance.

**Table 1:** Effect of NR3C1 ligands on 2×GRE activation in the presence and absence of TNF.

	Naive		+TNF	
	*E*_max_ (% Dex)	pEC_50_ (M)	*E*_max_ (% Dex)	pEC_50_ (M)
Dexamethasone	100 ± 3.2	7.6 ± 0.1	58.7 ± 3.2	7.5 ± 0.1 *
Fluticasone Propionate	111.2 ± 4.3	8.2 ± 0.1	51.0 ± 2.8	8.2 ± 0.1
Budesonide	109.9 ± 2.6	8.2 ± 0.1	56.9 ± 2.0	8.1 ± 0.1
Fluticasone Furoate	108.0 ± 2.9	8.8 ± 0.0	57.8 ± 1.5	8.7 ± 0.0
GSK9027	77.2 ± 1.0	7.1 ± 0.0	40.7 ± 0.9	7.0 ± 0.1
Des-ciclesonide	76.0 ± 2.0	8.6 ± 0.1	43.1 ± 1.0	8.5 ± 0.1
RU24858	74.9 ± 1.1	7.4 ± 0.0	46.0 ± 0.8	7.3 ± 0.0 *
GW870086X	50.1 ± 1.1	8.3 ± 0.1	29.8 ± 1.4	8.0 ± 0.2 *

BEAS-2B cells harboring a 2×GRE reporter were either not treated (Naive) or were pre-treated with tumor necrosis factor (TNF; 10 ng/ml) for 1 h, prior to addition of a maximally effective concentration of the indicated NR3C1 ligands. After 6 h, cells were harvested for luciferase assay. Data are from Fig. 5 and are expressed as a percent of 1 μM dexamethasone treatment (Naïve: *E*_Max_ = 6.3 ± 0.2 fold; +TNF *E*_Max_ = 3.7 ± 0.2 fold). Statistical analyses were conducted by Student’s t test to compare the EC_50_s of the ligand in the presence and absence of TNF, as indicated: P < 0.05 *.

**Fig 6:**
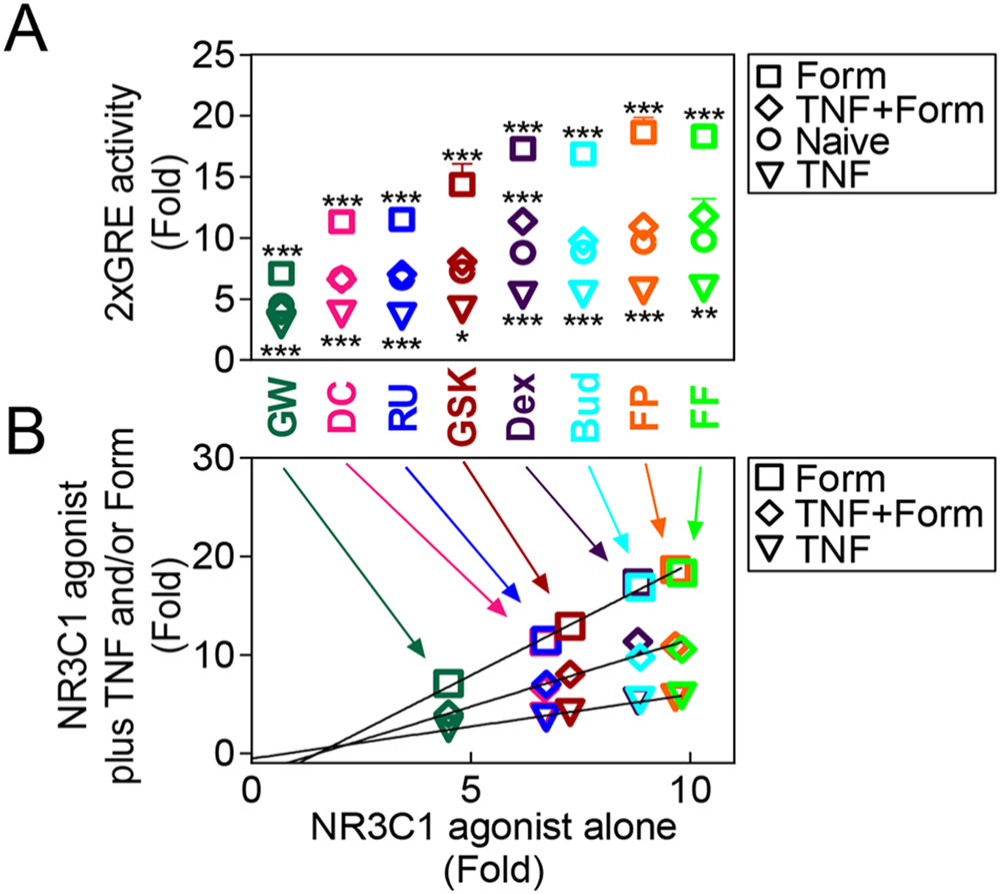
Effects of TNF and formoterol on 2×GRE activation induced by NR3C1 ligands. BEAS-2B 2×GRE cells were pre-treated with 10 ng/ml of tumor necrosis factor-α (TNF) for 1 h, before addition of maximally effective concentrations of fluticasone furoate (FF; 100 nM), fluticasone propionate (FP; 100 nM), budesonide (Bud; 100 nM), dexamethasone (Dex; 1 μM), GSK9027 (GSK; 1 μM), RU24858 (RU; 1 μM), des-ciclesonide (DC; 100 nM), GW870086X (GW; 100 nM), in the presence or absence of 10 nM formoterol (Form). Cells were harvested after 6 h for luciferase assays. Data (n = 7), expressed as fold activation, are plotted as means ± S.E. Lines of best fit were added in panel B. Statistical analyses were performed by ANOVA with a Dunnett’s test comparing each NR3C1 ligand alone versus in the presence of TNF and/or Form. *, *P*<0.05; **, *P*<0.01; ***, *P*<0.001.

The experiments in Fig. 5 were performed on different occasions and, as a consequence, differences in the levels of TNF-dependent repression were noted for the reference agonist, dexamethasone. To directly compare the repressive effect of TNF on each ligand, 2GRE reporter cells were treated with a maximally effective concentration of each agonist in the absence, or presence, of TNF (Fig. 6). In each case, TNF significantly reduced reporter activity by 44–55% (Fig. 6A). Thus, a plot of fold activation for each ligand against the fold activation in the presence of TNF was linear (*r* ^2^ = 0.71) (Fig. 6B), i.e. the level of reporter activation achieved in the presence of TNF was proportional to the *E*_Max_ produced by each ligand in the absence of TNF.

LABAs, such as formoterol, can synergistically enhance the transcriptional activity of glucocorticoids [23]. We therefore investigated the effect of a maximally effective concentration (10 nM) of formoterol, alone and in the presence of TNF, on 2×GRE reporter activity induced by each NR3C1 ligand (Fig. 6). Reporter activity induced by each ligand was significantly potentiated by ~2 fold by formoterol. Plotting the fold activation produced by NR3C1 ligand plus formoterol against the fold activation for each ligand alone revealed a linear relationship (*r* ^*2*^ = 0.72) (Fig. 6B). In the presence of TNF, formoterol restored 2×GRE reporter activity induced by each NR3C1 ligand to, or close to, the level of activation produced by the NR3C1 ligand alone (Fig. 6A & B). Thus, repression of GRE-dependent transcription by TNF and the enhancement by formoterol depend on the original *E*_Max_ for each ligand and together, at equivalent ligand concentrations, the effects of TNF and formoterol act to virtually cancel each other out.

### Effects of Inhibitors of the JNK and NF-Κb Pathways on Gene Expression

**Fig 7:**
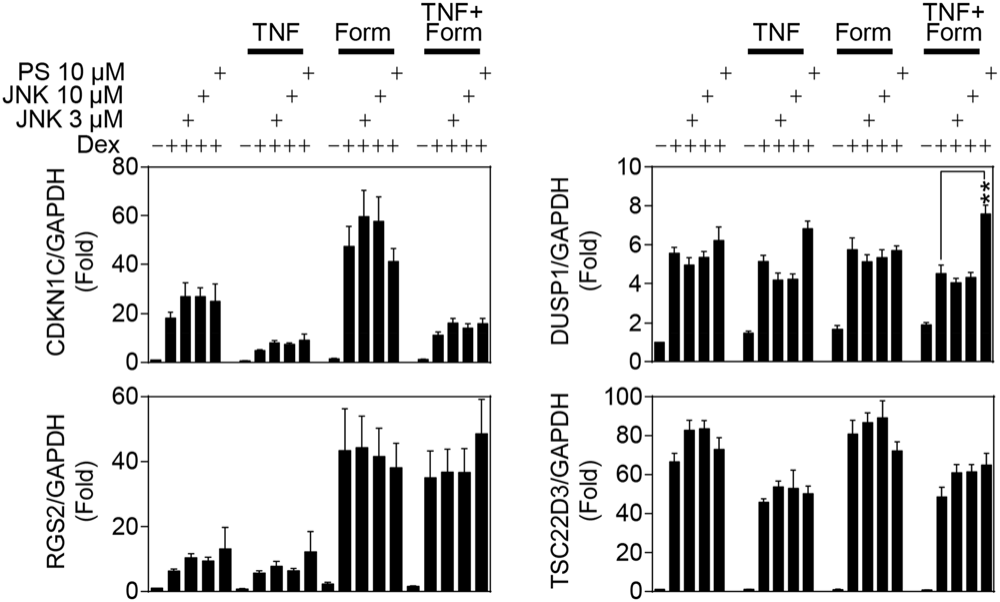
Effects of JNK MAPK or IKK2 inhibition on TNF-induced repression of glucocorticoid inducible gene expression. BEAS-2B cells were pre-treated for 30 min with either 10 or 3 μM of the JNK MAPK inhibitor JNK inhibitor VIII (JNK) or 10 μM of the IKK2 inhibitor PS-1145 (PS), before addition of 10 ng/ml tumor necrosis factor-α (TNF). After 1 h, 1 μM dexamethasone (Dex) and/or 10 nM formoterol (Form) was added and cells were harvested 6 h later. Total RNA was extracted, cDNA synthesized and RT-PCR performed for: cyclin-dependent kinase inhibitor 1C (CDKN1C; p57KIP2), dual specificity phosphatase 1 (DUSP1; MKP1), regulator of G-protein signaling 2 (RGS2), TSC22 domain family member 3 (TSC22D3; GILZ) and glyceraldehyde-3-phosphate dehydrogenase (GAPDH). Data (n = 9), normalized to GAPDH, are expressed as fold and plotted as means ± S.E. Significance was tested using repeated measures, one-way analysis of variance (ANOVA) with Bonferroni’s correction for multiple comparisons. **, *P*<0.01. Dexamethasone significantly increased expression of all four genes.

To test the effect of IKK2 or JNK inhibition of TNF-induced repression of *bona fides* glucocorticoid inducible genes, BEAS-2B cells were pre-treated with PS-1145 or JNK inhibitor VIII for 30 min prior to TNF addition. After 1 h, dexamethasone and/or formoterol were added and the cells harvested 6 h later for gene expression analysis (Fig. 7). TNF and formoterol, either alone or in combination, had no significant effect on the expression of CDKN1C, DUSP1, RGS2 or TSC22D3. TNF pre-treatment repressed the ability of dexamethasone to induce TSC22D3 and CDKN1C mRNA expression, but, as in Fig. 2, did not substantially affect the dexamethasone-induced expression of RGS2 or DUSP1 mRNA. Addition of JNK inhibitor VIII (at 3 or 10 μM) to dexamethasone or dexamethasone plus TNF, had no significant effect on the mRNA expression of any gene tested. Likewise, PS-1145 (10 μM) had no significant effect on dexamethasone-induced mRNA expression of any of these four mRNAs, either in the absence, or presence, of TNF.

Formoterol did not substantially affect dexamethasone-induced mRNA expression of TSC22D3 or DUSP1, but significantly enhanced CDKN1C and RGS2 mRNA induction (Fig. 7). Addition of JNK inhibitor VIII had no significant effect on mRNA expression of these four genes when induced by dexamethasone plus formoterol. Likewise, PS-1145 had no effect on the expression of CDKN1C, RGS2 or TSC22D3 in the presence of dexamethasone plus formoterol or with the further addition of TNF (Fig. 7). However, addition of PS-1145 to dexamethasone plus TNF plus formoterol significantly enhanced DUSP1 mRNA expression and this is consistent with the trend towards enhanced DUSP1 expression following PS-1145 addition in the presence of dexamethasone plus TNF. Nevertheless, despite the effect of PS-1145 on DUSP1 expression, the inhibition of IKK2 or JNK did not significantly alter expression of these genes when induced by glucocorticoid or glucocorticoid in the presence of TNF.

### Effects of TNF and Formoterol on Gene Expression Induced by NR3C1 Ligands

**Fig 8:**
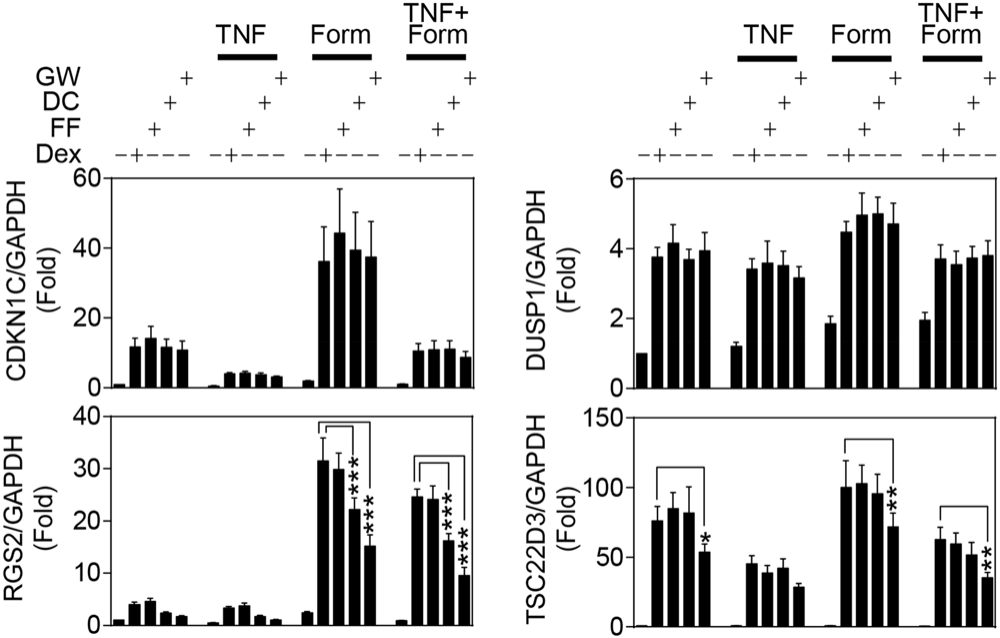
Effects of TNF and formoterol on gene expression induced by NR3C1 ligands. BEAS-2B cells were pre-treated for 1 h with 10 ng/ml of tumor necrosis factor-α (TNF), prior to the addition of 10 nM formoterol (Form) and/or the NR3C1 ligands: 1 μM dexamethasone (Dex), 100 nM fluticasone furoate (FF), 100 nM des-ciclesonide (DC) or 100 nM GW870086X (GW). Cells were harvested 6 h after NR3C1 ligand addition. Total RNA was extracted, cDNA synthesized and RT-PCR performed for: cyclin-dependent kinase inhibitor 1C (CDKN1C; p57KIP2), dual specificity phosphatase 1 (DUSP1; MKP1), regulator of G-protein signaling 2 (RGS2), TSC22 domain family member 3 (TSC22D3; GILZ) and glyceraldehyde-3-phosphate dehydrogenase (GAPDH). Data (n = 5), normalized to GAPDH, are expressed as fold and plotted as means ± S.E. Statistical significance was tested using repeated measures, one-way analysis of variance (ANOVA) with Bonferroni’s correction for multiple comparisons. *, *P*<0.05; **, *P*<0.01; ***, *P*<0.001. Each NR3C1 ligand significantly increased expression of all four genes.

The effect of NR3C1 ligands, showing full or various levels of partial agonism, were examined on the mRNA expression of CDKN1C, DUSP1, RGS2 and TSC22D3 in the presence of TNF and/or formoterol (Fig. 8). TNF and formoterol, alone or in combination, had no significant effect on the induction of any gene tested. Dexamethasone, fluticasone furoate, des-ciclesonide and GW870086X significantly induced the mRNA expression of all four genes. However, whereas all four agonists had an equivalent ability to promote CDKN1C or DUSP1 expression, there was a clear trend towards reduced mRNA induction, relative to the effect of dexamethasone, of RGS2 by both des-ciclesonide and GW870086X. Likewise, TSC22D3 mRNA expression was induced to a similar level by dexamethasone, fluticasone furoate and des-ciclesonide with fold inductions of 66, 73 and 71 respectively, while GW870086X produced a significantly lower, 50 fold activation (Fig. 8).

TNF pre-treatment significantly repressed CDKN1C and TSC22D3 expression induced by all four NR3C1 ligands, but did not affect ligand-induced RGS2 or DUSP1 expression. Formoterol enhanced dexamethasone-induced mRNA expression of all four genes, although only very modestly for DUSP1 and TSC22D3. In the presence of formoterol, the four NR3C1 ligands showed a similar ability to induce CDKN1C and DUSP1 expression. However, GW870086X in the presence of formoterol, or TNF plus formoterol, induced significantly lower TSC22D3 mRNA expression relative to dexamethasone. Likewise, the level of RGS2 mRNA expression induced in the presence of formoterol was dependent on the efficacy of the NR3C1 agonist, with both des-ciclesonide and GW87008X inducing significantly lower mRNA expression than dexamethasone (Fig. 8). These differences were also maintained in the presence of TNF plus formoterol. Thus, the level of mRNA expression achieved by different NR3C1 ligands is dependent on the agonist efficacy for some genes, but not others. However, the repressive effects of TNF and the enhancements produced by formoterol occurred irrespective of the agonist that was used.

## Discussion

Glucocorticoid activity can be repressed by numerous inflammatory cytokines, including IL2, IL4, IL13, IL17, IL23, interferon γ, transforming growth factor beta 1, IL1B and TNF, indicating that glucocorticoid resistance is likely to occur via multiple mechanisms [6,7,11,13,48–52]. In the current study, we focused on TNF-induced glucocorticoid resistance, as TNF expression is increased in asthmatic lungs and correlates with disease severity [53–55]. Indeed, TNF inhalation may increase eosinophil and neutrophil recruitment to the lung and enhance the sensitivity of the airways to methacholine [56,57]. Moreover, anti-TNF therapies, including etanercept, are reported to reduce symptoms and improve lung function in some severe asthmatics [53–55]. Importantly, by inducing core inflammatory signaling cascades, for example NF-κB and MAPKs, TNF promotes many effects in common with toll-like receptors and other pro-inflammatory cytokines, including IL1B and IL-33. Therefore, the current findings may have general applicability with respect to reduced glucocorticoid function in inflammation.

We now show that the repression of glucocorticoid-inducible gene expression by the pro-inflammatory cytokines TNF and IL1B occurs in primary human ASM and HBE cells. This is consistent with the attenuation of glucocorticoid-dependent transcription in human pulmonary A549 and bronchial epithelial BEAS-2B cells and collectively suggests that loss of glucocorticoid-induced gene expression may be a generic response to pro-inflammatory mediators [6]. Moreover, while it is apparent that IL1B reduces the expression of most dexamethasone-inducible genes [58], the current study shows that repression varies in a gene-, cell- and time-dependent manner. For example, TNF, or IL1B, pre-treatment significantly reduced dexamethasone-induced TSC22D3 expression at 2 h in ASM, but not HBE, cells. Likewise, RGS2 expression was modestly repressed at 6 h in HBE, but not BEAS-2B cells. Additionally, while there was no repression of dexamethasone-induced TSC22D3 in HBE cells at 2 h, repression by TNF or IL1B occurred at 1 and 6 h and was maintained at 18 h. In contrast, RGS2 mRNA expression was induced by dexamethasone at 1, 2, 6 and 18 h, but only showed repression by TNF or IL1B at 1, 2 and 6 h. Conversely, glucocorticoid-induced DUSP1 mRNA expression was unaffected by cytokine pre-treatment in HBE, ASM or BEAS-2B cells and this is consistent with DUSP1 induction by both glucocorticoids and inflammatory stimuli, including IL1B and TNF [22,36,59].

Approaches to overcoming TNF-induced glucocorticoid hyporesponsiveness were investigated using a 2×GRE reporter system. TNF activates numerous signaling pathways including PI3K, PKC, MAPK and NF-κB [60]. Indeed, PI3K inhibition reduced glucocorticoid resistance induced by IL-17A [51], MIP-2 [13], LPS, hydrogen peroxide or cigarette smoke [40,41]. However, in the present study, while three PI3K inhibitors all decreased AKT1 phosphorylation, they failed to reverse the repressive effect of TNF on the 2×GRE reporter. Likewise, the PKC inhibitors Ro 31–8220 and Gö6976 concentration-dependently repressed 2×GRE activation, while GF109203X enhanced reporter activation in the presence of dexamethasone alone or with TNF. Given that Ro 31–8220 is a relatively generic inhibitor of PKC isoforms, while Gö6976 and GF109203X are more selective for classical or classical and novel PKC isoforms [61], respectively, these data do not support a major role for PKC in the TNF-dependent glucocorticoid hyporesponsiveness.

Inhibition of p38 and ERK MAPK can reverse glucocorticoid resistance induced by TNF or IL2 plus IL4 in human keratinocyte and peripheral blood mononuclear cells, respectively [11,62]. However, in the current study, a p38 MAPK inhibitor, SB203580, did not alter the repressive effect of TNF on the 2×GRE reporter. Equally, an inhibitor of the ERK MAPK pathway, PD098059, significantly repressed dexamethasone-induced 2×GRE activation. Conversely, an inhibitor of JNK, JNK inhibitor VIII, significantly attenuated the ability of TNF to reduce 2×GRE activation. While the mechanisms underlying this effect are unclear, preventing the JNK-dependent phosphorylation of NR3C1, events which can reduce GR activity, could contribute [63–66]. The IKK2 inhibitor, PS-1145, also partially reversed the repressive effect of TNF on dexamethasone-induced 2×GRE reporter activity. However, the simultaneous addition of PS-1145 and JNK inhibitor VIII failed to produce any additional effect, possibly due to crosstalk between the NF-κB and JNK MAPK pathways induced by TNF [67].

Contrary to effects on the 2×GRE reporter, inhibition of IKK2 or JNK had no effect on the repression of dexamethasone-induced expression of CDKN1C and TSC22D3 by TNF in BEAS-2B cells. Rather, PS-1145 enhanced DUSP1 mRNA expression following treatment with dexamethasone plus formoterol in the presence of TNF, with a similar trend apparent with dexamethasone plus TNF. This is suggestive of a negative IKK2-dependent regulation, and indeed increased DUSP1 expression could result from enhanced MAPK activation due to repression of NF-κB [67]. However, given the clear attenuation of TNF-induced resistance on the 2×GRE reporter, we predict that genes showing glucocorticoid inducibility with repression by TNF that is reduced by IKK2 or JNK inhibition will exist. Furthermore, the NF-κB and JNK MAPK pathways are activated by multiple inflammatory stimuli and their inhibition is likely to be independently beneficial by reducing inflammatory mediator expression. As well as affecting inflammatory responses, this may alleviate concomitant loss of NR3C1 function.

While a more effective approach to overcoming glucocorticoid resistance may rest with agonists that are unaffected by cytokine pre-treatment, all the NR3C1 ligands, whether partial, full, steroidal or non-steroidal, that were tested, appeared equally vulnerable to TNF-induced loss of activity. Thus, fluticasone furoate, fluticasone propionate and budesonide induced 2×GRE reporter activity to a similar level as dexamethasone and are considered as full agonists. Conversely, GSK9027, RU24858, GW870086X and des-ciclesonide all demonstrated reduced 2×GRE reporter activation and are partial agonists. Nevertheless, in the presence of TNF, reporter activity was reduced by a similar fraction for each ligand. Thus the full agonists demonstrated a greater ability to induce 2×GRE activation and this superiority was maintained even after TNF pre-treatment. Therefore, if symptom control in asthmatics depends on gene expression that behaves like the simple 2×GRE reporter, then developing higher efficacy agonists may be more effective in the context of glucocorticoid resistance. However, marked differences between effects on the simple GRE reporter and real genes were observed. Thus, RGS2 mRNA expression was highly dependent on NR3C1 ligand efficacy. Similarly, TSC22D3 expression was strongly induced by fluticasone furoate, dexamethasone and des-ciclesonide, but showed reduced inducibility by GW870086X. Conversely, CDKN1C and DUSP1 expression was induced to similar levels by full and partial agonists. Consequently full agonists may produce maximal biological effects in respect of each of these genes, whereas partial agonists, for example GW870086X, may show diminished functional effects due to reduced RGS2 and TSC22D3 expression. Given a gene- and cell-dependence, such effects require specific testing in relevant target tissues. If a low efficacy agonist was a partial agonist for RGS2 expression in airway smooth muscle, one might predict reduced levels of bronchoprotection [26,27,29]. Therefore, if the beneficial activities of glucocorticoids require RGS2 and TSC22D3 expression, weaker agonists may prove suboptimal. Conversely, if genes responsible for side effects are induced in a ligand efficacy-dependent manner, SEGRAs with reduced gene expression profiles may show fewer side effects. This will be influenced by the level and isoform of NR3C1 expressed in target tissues [68,69], as well as by delivery route. Hence full agonists may be optimal for inhaled delivery to the lung, where side-effects are a lesser issue, whereas, partial agonists, for example GW870086X, may be more suited to systemic delivery where metabolic and endocrine effects are a concern [19,70]. However, although RU24858 has some separation between transactivation and repression *in vitro* [20,21], these effects are diminished *in vivo* and do not appear to translate into a reduced side effect profile [71]. Nevertheless, ligands such as GW870086X, which was a weaker agonist relative to RU24858, shows a reduced side effect profile [19,70].

Finally, the most effective approach to overcoming TNF-induced glucocorticoid hyporesponsiveness was the concurrent addition of formoterol. This countered the repressive effects of TNF on both the 2×GRE reporter and glucocorticoid-induced gene expression and restored glucocorticoid activity to that achieved in the absence of TNF. Indeed, LABAs improve symptom control in severe asthma and during exacerbations and are therefore the recommended step-up therapy for moderate and severe asthmatics not responding to ICS monotherapy [68]. While the mechanisms by which LABAs enhance glucocorticoid activity are not fully understood [68], the enhancement NR3C1 transactivation by formoterol was also dependent on ligand efficacy. Thus combination of a high efficacy ICS (such as fluticasone furoate, fluticasone propionate or budesonide) with LABA may provide superior benefit compared to combination of a LABA with a lower efficacy ICS (e.g. des-ciclesonide), in poorly controlled asthma. In conclusion, cytokines repress glucocorticoid-inducible gene expression in multiple cell types relevant to asthma and COPD. This effect was not surmountable with any glucocorticoid tested. While TNF-induced glucocorticoid hyporesponsiveness was partially reversed on a simple GRE reporter following inhibition of IKK2 or JNK, this did not translate to effects at the level of gene expression and therefore benefits, aside from reducing inflammatory gene expression, are unclear. By enhancing glucocorticoid activity, formoterol was the most effective means to functionally reverse TNF-induced glucocorticoid resistance. These data suggest that the addition of LABAs to higher efficacy NR3C1 ligands may represent a superior means to treat steroid-resistant inflammatory conditions such as severe asthma and COPD.

## Supporting Information

S1 Fig PS1145, an IKK2 inhibitor, and JNK inhibitor VIII concentration-dependently inhibit phosphorylation of NFKBIA and JUN respectively. **A**. Human bronchial epithelial, BEAS-2B, cells were pre-treated for 30 min with the indicated concentrations of PS-1145 (PS), before addition of tumor necrosis factor-α (TNF). Cells were harvested after 2 min for western blotting and probed for phospho nuclear factor of kappa light polypeptide gene enhancer in B-cells inhibitor alpha (pNFKBIA; pIκBα), NFKBIA and glyceraldehyde 3-phosphate dehydrogenase (GAPDH). **B**. BEAS-2B cells were pre-treated for 30 min with the indicated concentrations of JNK inhibitor VIII (JNK), before addition of 10 ng/ml TNF. Cells were harvested after 15 min for western blotting and probed for phospho jun proto-oncogene (pJUN) and GAPDH.
(PDF)

S2 Fig PKC inhibitors concentration-dependently inhibit AKT1 phosphorylation. Human bronchial epithelial, BEAS-2B, cells were pre-treated for 30 min with the indicated concentrations of **A**. LY294002 (LY), **B**. PI103 (PI) or **C**. wortmannin (W), before addition of 10 ng/ml tumor necrosis factor-α (TNF). Cells were harvested after 30 min for western blotting and probed for phospho v-akt murine thymoma viral oncogene homolog 1 (pAKT1), AKT1 and glyceraldehyde 3-phosphate dehydrogenase (GAPDH).
(PDF)
