# Correction: Cytokine-Induced Loss of Glucocorticoid Function: Effect of Kinase Inhibitors, Long-Acting β2-Adrenoceptor Agonist and Glucocorticoid Receptor Ligands

**DOI:** 10.1371/journal.pone.0128728

**Published:** 2015-05-14

**Authors:** The PLOS ONE Staff

There is an error in the title. The correct title is: Cytokine-Induced Loss of Glucocorticoid Function: Effect of Kinase Inhibitors, Long-Acting β_2_-Adrenoceptor Agonist and Glucocorticoid Receptor Ligands. The correct citation is: Rider CF, Shah S, Miller-Larsson A, Giembycz MA, Newton R (2015) Cytokine-Induced Loss of Glucocorticoid Function: Effect of Kinase Inhibitors, Long-Acting β_2_-Adrenoceptor Agonist and Glucocorticoid Receptor Ligands. PLoS ONE 10(1): e0116773. doi:10.1371/journal.pone.0116773

There is an error in the title of the second subsection of the Results. The correct title is: Effect of MAPK and NF-κB Inhibitors on TNF-Induced Glucocorticoid Resistance.

There is an error in the second sentence of the second paragraph of the “Effect of Different NR3C1 Ligands and Formoterol on TNF-Induced Glucocorticoid Resistance” subsection of the Results. The correct sentence is: To directly compare the repressive effect of TNF on each ligand, 2×GRE reporter cells were treated with a maximally effective concentration of each agonist in the absence, or presence, of TNF (Fig. 6).

There is an error in the title of the fifth subsection of the Results. The correct title is: Effects of Inhibitors of the JNK and NF-κB Pathways on Gene Expression.

The publisher apologizes for the errors.
