# Supplementary material for: Cytokine-Induced Loss of Glucocorticoid Function: Effect of Kinase Inhibitors, Long-Acting β2-Adrenoceptor Agonist and Glucocorticoid Receptor Ligands
Source: PLoS One. 2015 Jan 27;10(1):e0116773. doi: 10.1371/journal.pone.0116773 (PMC4308083; doi:10.1371/journal.pone.0116773)
Supplement: S1 Fig — A. Human bronchial epithelial, BEAS-2B, cells were pre-treated for 30 min with the indicated concentrations of PS-1145 (PS), before addition of tumor necrosis factor-α (TNF). Cells were harvested after 2 min for western blotting and probed for phospho nuclear factor of kappa light polypeptide gene enhancer in B-cells inhibitor alpha (pNFKBIA; pIκBα), NFKBIA and glyceraldehyde 3-phosphate dehydrogenase (GAPDH). B. BEAS-2B cells were pre-treated for 30 min with the indicated concentrations of JNK inhibitor VIII (JNK), before addition of 10 ng/ml TNF. Cells were harvested after 15 min for western blotting and probed for phospho jun proto-oncogene (pJUN) and GAPDH. (PDF) [file pone.0116773.s001.pdf]

## Supporting Figure S1

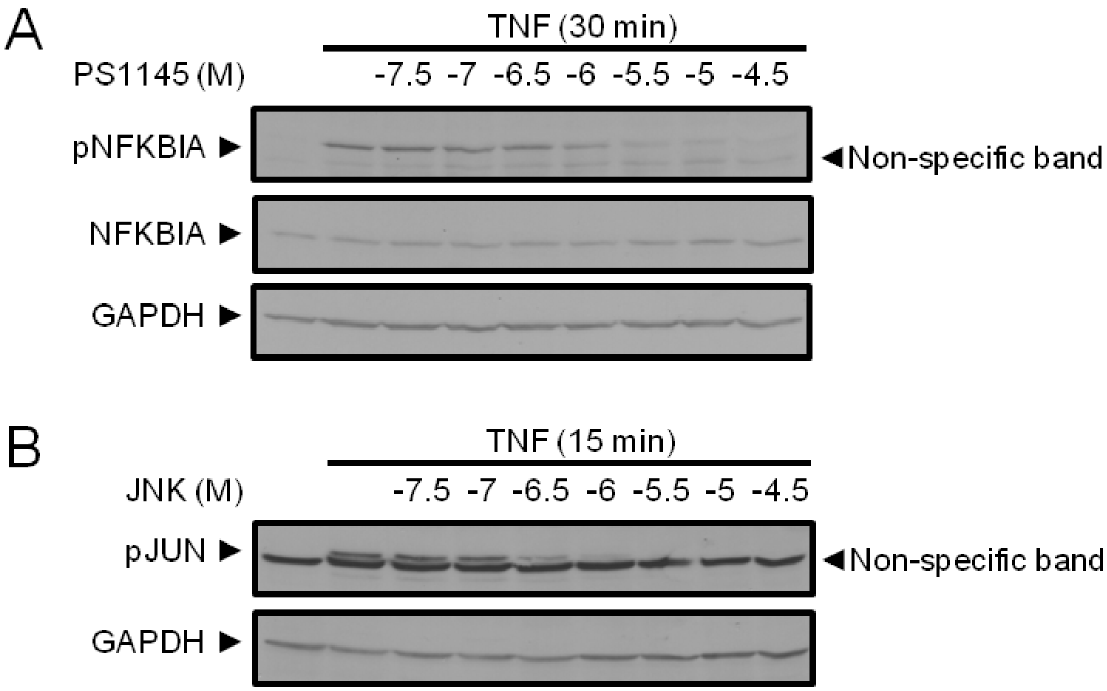


**Supporting Figure S1. PS1145, an IKK2 inhibitor, and JNK inhibitor VIII concentration-dependently inhibit phosphorylation of NFKBIA and JUN respectively**

**A**. Human bronchial epithelial, BEAS-2B, cells were pre-treated for 30 min with the indicated concentrations of PS-1145 (PS), before addition of tumor necrosis factor-α (TNF). Cells were harvested after 2 min for western blotting and probed for phospho nuclear factor of kappa light polypeptide gene enhancer in B-cells inhibitor alpha (pNFKBIA; pIκBα), NFKBIA and GAPDH. **B**. BEAS-2B cells were pre-treated for 30 min with the indicated concentrations of JNK inhibitor VIII (JNK), before addition of 10 ng/ml TNF. Cells were harvested after 15 min for western blotting and probed for phospho jun proto-oncogene (pJUN) and glyceraldehyde 3-phosphate dehydrogenase (GAPDH).
