# Supplementary material for: Cytokine-Induced Loss of Glucocorticoid Function: Effect of Kinase Inhibitors, Long-Acting β2-Adrenoceptor Agonist and Glucocorticoid Receptor Ligands
Source: PLoS One. 2015 Jan 27;10(1):e0116773. doi: 10.1371/journal.pone.0116773 (PMC4308083; doi:10.1371/journal.pone.0116773)
Supplement: S2 Fig — Human bronchial epithelial, BEAS-2B, cells were pre-treated for 30 min with the indicated concentrations of A. LY294002 (LY), B. PI103 (PI) or C. wortmannin (W), before addition of 10 ng/ml tumor necrosis factor-α (TNF). Cells were harvested after 30 min for western blotting and probed for phospho v-akt murine thymoma viral oncogene homolog 1 (pAKT1), AKT1 and glyceraldehyde 3-phosphate dehydrogenase (GAPDH). (PDF) [file pone.0116773.s002.pdf]

Supporting Figure S2

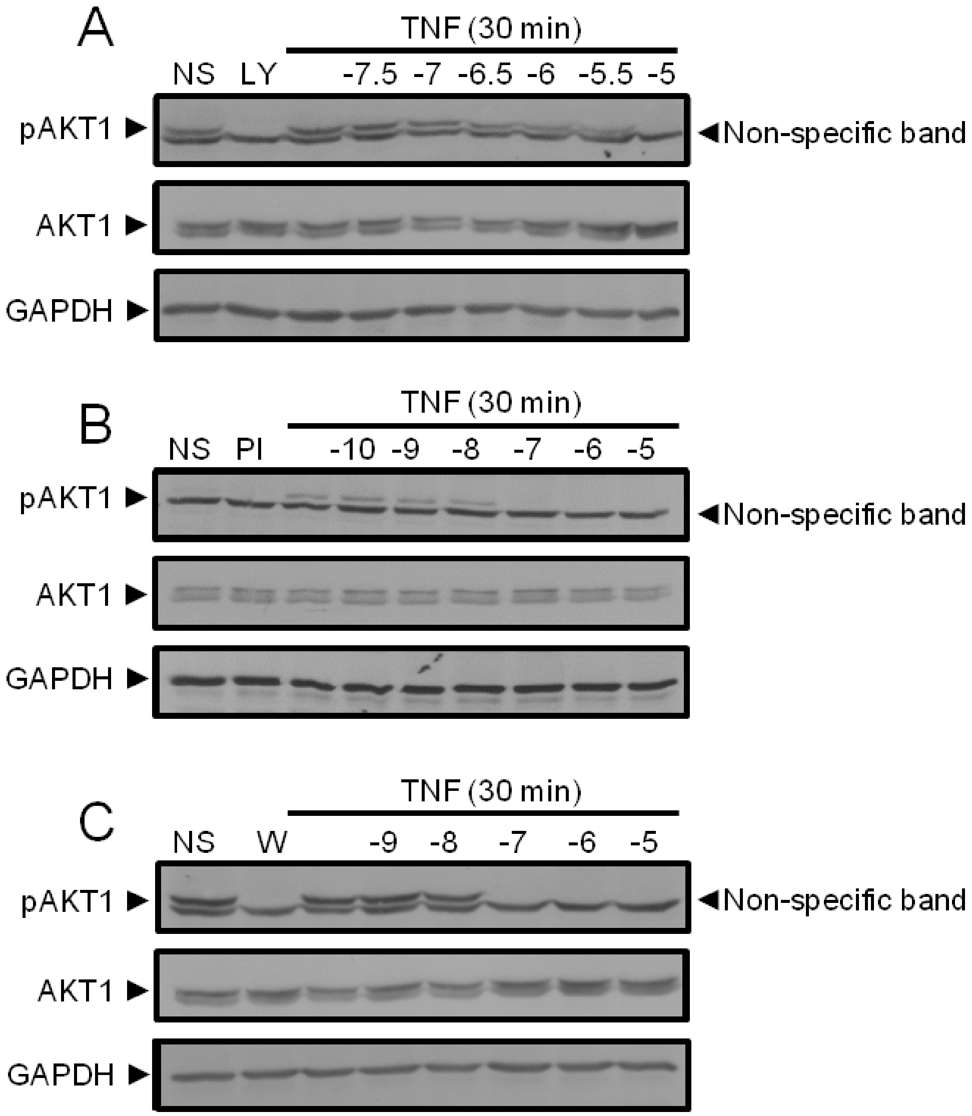

Supporting Figure S2. PKC inhibitors concentration-dependently inhibit AKT1 phosphorylation

Human bronchial epithelial, BEAS-2B, cells were pre-treated for 30 min with the indicated concentrations of **A**. LY294002 (LY), **B**. PI103 (PI) or **C**. wortmannin (W), before addition of 10 ng/ml tumor necrosis factor-α (TNF). Cells were harvested after 30 min for western blotting and probed for phospho v-akt murine thymoma viral oncogene homolog 1 (pAKT1), AKT1 and glyceraldehyde 3-phosphate dehydrogenase (GAPDH).
